# Supplementary material for: Cooperative Effect of Ni-Decorated Monolayer WS2, NiO, and AC on Improving the Flame Retardancy and Mechanical Property of Polypropylene Blends
Source: Polymers (Basel). 2023 Jun 23;15(13):2791. doi: 10.3390/polym15132791 (PMC10347200; doi:10.3390/polym15132791)
Supplement: Supplementary file 1 [file polymers-15-02791-s001.zip › polymers-2391987-supplementary.pdf]

Cooperative Effect of Ni-Decorated Monolayer WS_2, NiO, and AC on Improving the Flame Retardancy and Mechanical Property of Polypropylene Blends

Mingqiang Shao [1,*], Yiran Shi [1], Jiangtao Liu [1], Baoxia Xue [1] and Mei Niu [1,*]

1 College of Textile Engineering, Taiyuan University of Technology, Taiyuan 030024, China;
* Correspondence: shaomingqiang@tyut.edu.cn (M.S.); niumei@tyut.edu.cn (M.N.)

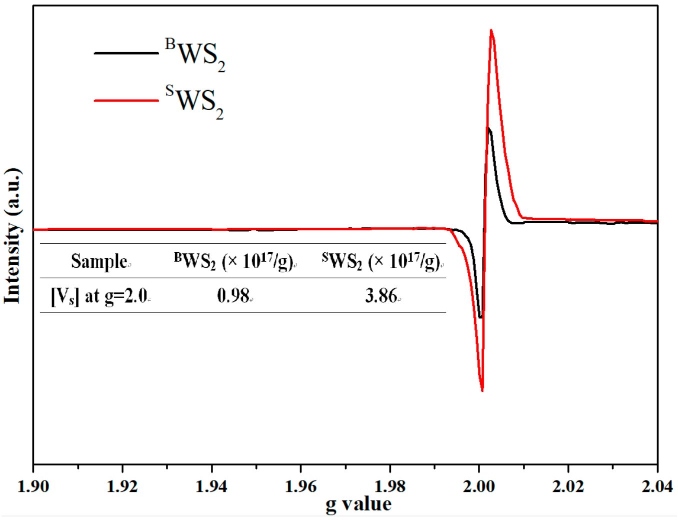

Figure S1. EPR spectra of bulk WS_2 ($^BWS_2$), and single layer WS_2 ($^SWS_2$), and the number of sulfur vacancies.

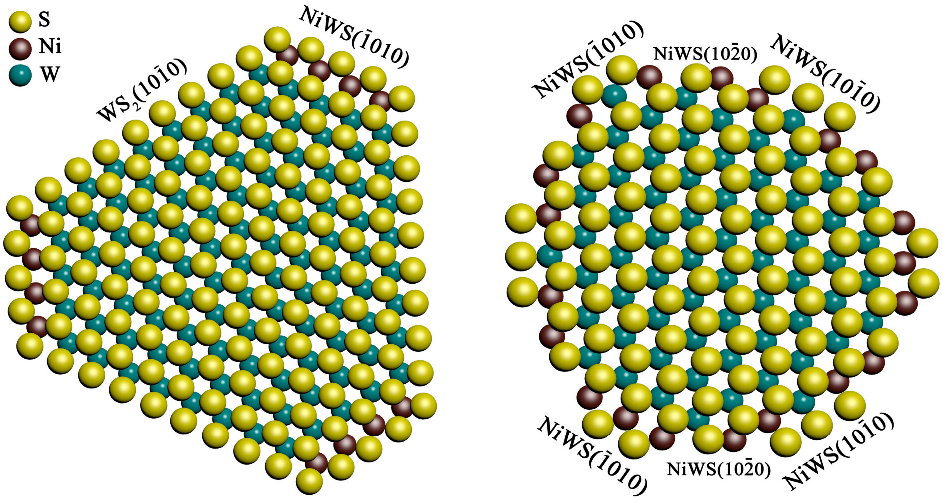

Figure S2. Scheme of typical Ni-W-S model.
